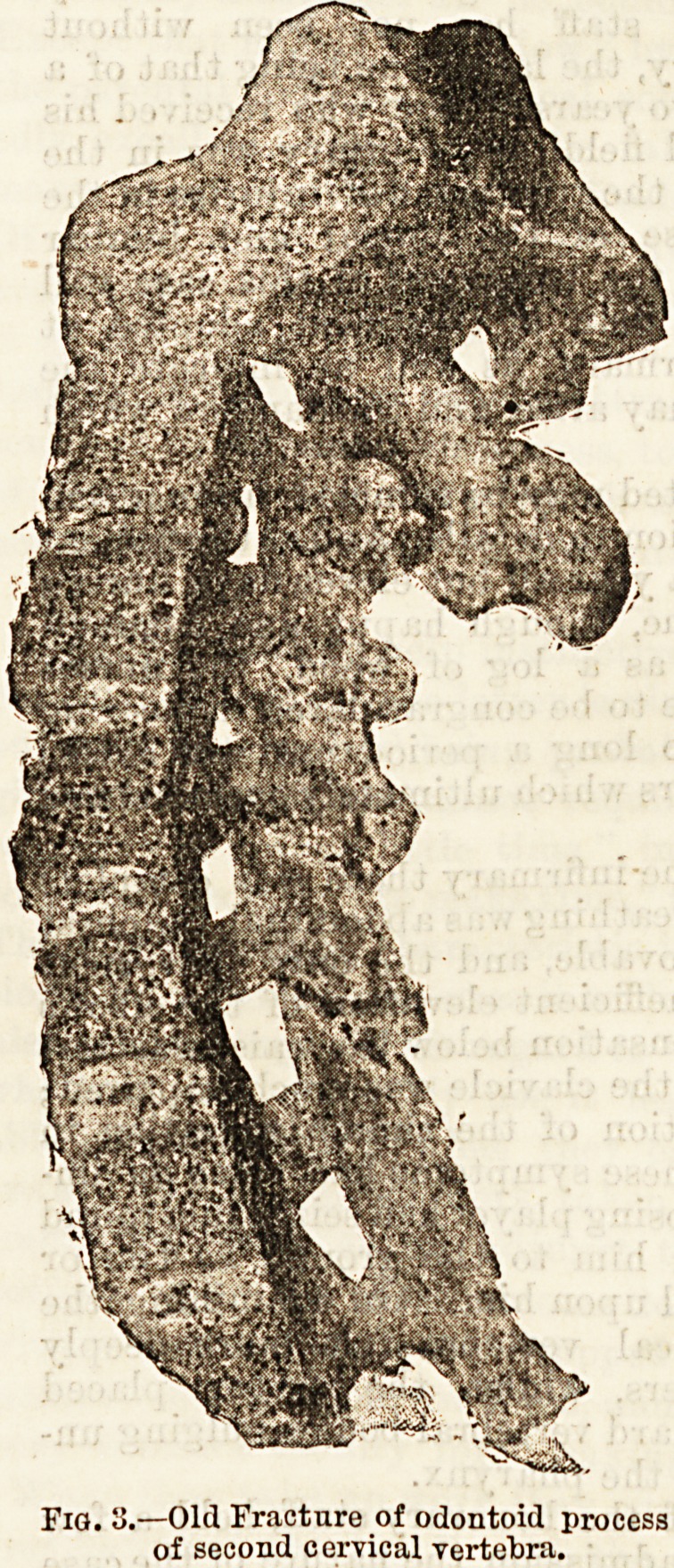# Fracture and Dislocation of the Spine

**Published:** 1893-09-23

**Authors:** Norman Porritt

**Affiliations:** Honorary Surgeon


### The Hospital Clinic.

[*The Editor will be glad to receive offers of co-operation and contributions from members of the profession. All letters should be addressed to* The Editor, The Lodge, Porchester Square, London, W.]

### HUDDERSFIELD INFIRMARY.

### Fracture and Dislocation of the Spine.

By Norman Porritt, M.R.C.S, L.R.C.P., Honorary Surgeon.

Fracture of the spine shares with fracture of the skull the unenviable distinction of belonging to the gravest and most dangerous class of fractures with which doctors and nurses have to deal. The reason is obvious. For the spine, like the cranium, is the storehouse and shield of delicate nervous structures, some essential to the continuance of life itself, and others just as necessary to the performance of some of the most important, if not absolutely vital, functions of the body. Any encroachment upon the spinal canal by fragments of broken bone or by a displaced vertebra may cause pressure upon the spinal cord or any degree of laceration of the cord from a slight tear to complete severance of the soft nervous structure. When the spinal cord is cut through its functions below the seat of the injury are at once annihilated, whilst pressure which does not sever the cord may be severe enough to have an exactly similar effect.

Fractures and dislocations of the spine are only produced by great violence, and in colliery districts, where pitmen are not unfrequently brought to hospital after being crushed by a fall of the roof of pit workings, they are by no means uncommon. Though the Huddersfield Infirmary serves a manufacturing rather than a colliery district, the surgical staff has not been without examples of this injury, the latest one being that of a young man, twenty-two years of age, who received his injury in the football field. The injury was in the cervical region, and as the treatment of injuries in the cervical includes those of the dorsal and lumbar regions, an outline of the treatment of this case will illustrate the general plan of treatment adopted at the Huddersfield Infirmary, as well as indicate the complications which may arise, and the dangers which have to be avoided.

The treatment adopted may be summed up in a sentence as a close attention to details. And as the patient survived his accident a year all but eleven days, during the whole of which time, though happy and comfortable, he was helpless as a log of wood, the nurses and house surgeons are to be congratulated in steering a hopeless case for so long a period past the shoals and through the dangers which ultimately overwhelmed him.

When admitted to the infirmary there was complete paralysis of the legs, breathing was abdominal, the chest being absolutely immovable, and the only movement in the arms was an inefficient elevation of the elbows from the bed. All sensation below the waist was lost and from the waist to the clavicle was much impaired; whilst, with the exception of the thumb, sensation in each arm was lost. These symptoms had come on immediately after an opposing player had seized him round the neck and dragged him to the ground, as two or three other players fell upon him. On examining the spine the sixth cervical vertebra felt more deeply placed than the others, whilst the finger placed in the mouth felt the hard vertebral bodies bulging unnaturally forward into the pharynx.

At a consultation of the honorary staff, held a few hours after the man's admission, the nature of the case was recognised and the line of treatment agreed upon.

1. *To Prevent Bedsores.*—The patient was laid upon a water-bed. The back was examined daily, carefully washed, then dried as thoroughly as possible by mopping with a towel and painted thickly with collodion. For seven weeks no bedsore formed. Then, in spite of every attention, the skin broke and the breach became a deep and wide irregular hole. The sore spread by sloughing when poultices and boracic fomentations encouraged the separation of the slough. Afterwards zinc ointment on lint or red lotion was applied; the sore filled up, and though it never healed, the raw surface was reduced by cicatrisation from an area of about twelve square inches to half that size.

On the powerless legs, and to a less extent also on the arms, sores formed, a result to which the œdema of the limbs contributed. By pillows and pads arranged under the legs pressure was taken from the threatening sores as much as possible, but the helpless legs fell over on their sides in spite of all the attempts to support them.

2. *To Guard against Retention of Urine and Consequent Cystitis.*—During the whole of the man's illness he never had cystitis. A soft rubber catheter was passed night and morning, and the urine having been drawn the bladder was washed out with warm boracic lotion. A three ounce syringe, supplied by Messrs. Down, having

Fig. 1.—Down's syringe with tap and nozzle to fit any catheter.

a pointed brass nozzle to fit any sized catheter, and provided with a tap, having been filled with the lotion, the nozzle was inserted into the end of the catheter, the tap turned, and the contents of the syringe were gently forced into the bladder; and the syringe being removed, the lotion flowed back into the vessel which had received the urine. This was repeated several times in order to thoroughly scour the bladder with the lotion at each operation. This plan is on the whole more convenient than the syphon irrigation, figured in text books, whilst it is unattended with the danger of over distending the bladder.

Sometimes the urine spurted out unknown to the patient. To keep him dry, an ordinary hospital male glass urinal was kept constantly in position, resting between and upon his thighs, and in this manner much of the wetting—which is so fruitful a cause of bedsores—was prevented. A sheet of wadding was placed between him and the drawsheet, and in it the nurse was able to remove the excretion from the bowel with as little trouble as possible, when an involuntary action took place. Constipation was the usual condition, fortunately, and the action of the bowels was timed and regulated with tolerable certainty by the administration of a simple enema.

3. *To Watch the Temperature.*—Injuries to the spine, and especially in the cervical region, are frequently followed by erratic extremes of temperature. In one case there may be hyperpyrexia; in another a-pyrexia. In the case which is the text of this article, six days after the injury the temperature reached 103·7 deg. to fall the next day till the thermometer registered as low as 90 deg. in six more days. At the same time the pulse and respiration rates dropped, the pulse at one time beating only 31, and the respirations numbering only seven and a half, per minute. Concurrently the surface of the body, not excluding the trunk and the parts protected by bedclothes, felt cold to the touch, and the patient was lethargic and inclined to wander. He was surrounded by hot bottles and covered with warm bedclothes, whilst stimulants and strong liquid food (which he took freely) were given; and five anxious days of a-pyrexia were followed by a welcome rise of temperature, and a return of the pulse and respiration to approximately natural rates. If hyper-pyrexia follows injury of the spine, quinine in large doses, Warburg's tincture, antifebrin, antipyrin, or phenacetene may in turn be tried, if with cold sponging, added to complete rest to the injured part, the excessive temperature cannot be controlled.

Fig. 2.—Section of spine showing dislocation of seventh cervical vertebra.

Another reason for keeping a watch over the temperature is that it is the danger signal of any local inflammatory action which may take place. No less than three attacks of cellular inflammation, erysipelatous in character, occurred around the bedsores of this patient, and to the last of those three attacks, ending as it did in hypostatic pneumonia, he succumbed. The tincture of perchloride of iron, in fifteen minim doses, with two grains of quinine in an ounce of water, given every four hours, was the medicine used, and the erysipelas was painted with collodion. The œdema of the loins and legs not only contributed to the formation of the sores, but provided a medium prone to take on cellular inflammation.

4. *Sweating.*—Excessive sweating was another troublesome symptom. Liq. atropia sulph. in one drop doses, with liq. strychniæ in five drop doses, seemed to have no influence whatever upon it.

5. *Local Treatment.*—This, the first phase of the case which the surgeon will have to consider may be discussed under three heads, viz.: Those of (1) absolute rest, (2) extension, (3) trephining.

*Absolute Rest.*—No other treatment is, in the great majority of cases, admissible; and it is one of the canons of surgery that excessive manipulation for the purposes of examination is in all cases to be avoided, lest the loose damaged bones be pressed further into the cord. The nature of the injury is to be inferred from the symptoms it produces—the paraplegia, the incontinence of urine, the loss of sensation—rather than from the signs at the seat of the injury. Not unfrequently the local signs are too insignificant to enable the surgeon to decide that a fracture has taken place. The patient should be placed upon a water bed, the spine being most carefully supported, immediately above and below the injury, whilst the patient is being lifted and placed upon his back, with the spine as nearly as possible in the normal position. If a water bed cannot be procured, a spring or hair mattress should take its place, a flock or feather bed being quite inadmissible. A fracture board, the size of the hair mattress, and placed underneath it, will maintain a firm, even surface for the support of the injured part.

*Extension.*—If the spine does not fall into its natural position when the patient is placed in the bed prepared for him; or if there is great pain at the seat of the injury, indicating nerve pressure, it is permissible to make a cautious trial of extension. It may be done with or without an anæsthetic. Whilst the trunk is held, powerful traction is made on each leg by assistants, the surgeon himself pressing upon the deformed part to force it into position if possible. If the manœuvre succeeds, and the injury is not above the middle of the dorsal region, a plaster jacket should be at once applied to keep the parts in position. When the injury is in the cervical region the crippled respiration almost contra-indicates the use of an anæsthetic, as few would take the responsibility of the administration when only the diaphragm is at work. Although powerful pressure was made upon the projection into the pharynx of the patient in the Huddersfield Infirmary, whilst the spine was extended by the legs and head, reduction could not be effected; and an extension apparatus made for the head and fitted with a weight and pulley, caused so much pain that it had to be discontinued after a day or two. No cases have yet been treated in the Huddersfield Infirmary by Sayre's extension apparatus, followed by the application of a plaster jacket, and the plan does not appear unattended with considerable risk.

*Trephining.*—More than one consultation of the staff was held to consider the advisability of cutting down upon the injured part, with the object of attempting to relieve the pressure upon the spinal cord. It was decided that no benefit was to be gained from the operation, and the result of the autopsy confirmed this view. A glance at figure 2 shows that the injury was one of dislocation of the seventh cervical vertebra. The spinal cord appeared as if it had been cut across by the displaced bone at the time of the accident. This case, therefore, tells against operative interference; and another case, which was in the Infirmary in 1875, teaches a similar lesson. In the latter case the patient fell a depth of two yards, alighting upon the back of his neck. He had no paralytic symptoms, but on getting up found he could not turn his head upon his shoulder, could not swallow solid food, his voice had acquired a peculiar nasal tone, he could not breathe through his nose, and on examining the pharynx an irregular projection was found close to the soft palate. These symptoms and signs persisted, the man went home, lived ten years afterwards, and by the kindness of Dr. Irving we are enabled to give a picture of the condition found on his death, a glance at which reveals a fracture of the odontoid process, with an impaction of the second into the third cervical vertebra, which were found to be united together by firm, bony anchylosis. In this case death was not due to the injury, the man being able to go about with the disadvantage only of a stiff neck. (*See Fig.* 3.)

## Figures and Tables

**Fig. 1. f1:**
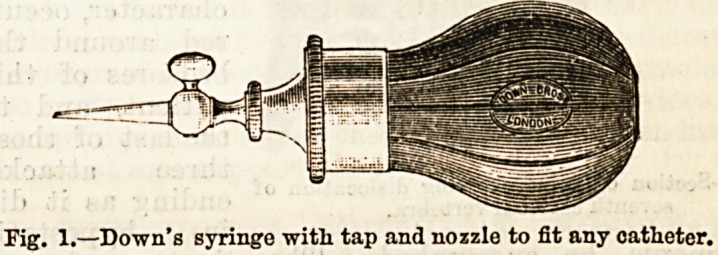


**Fig. 2. f2:**
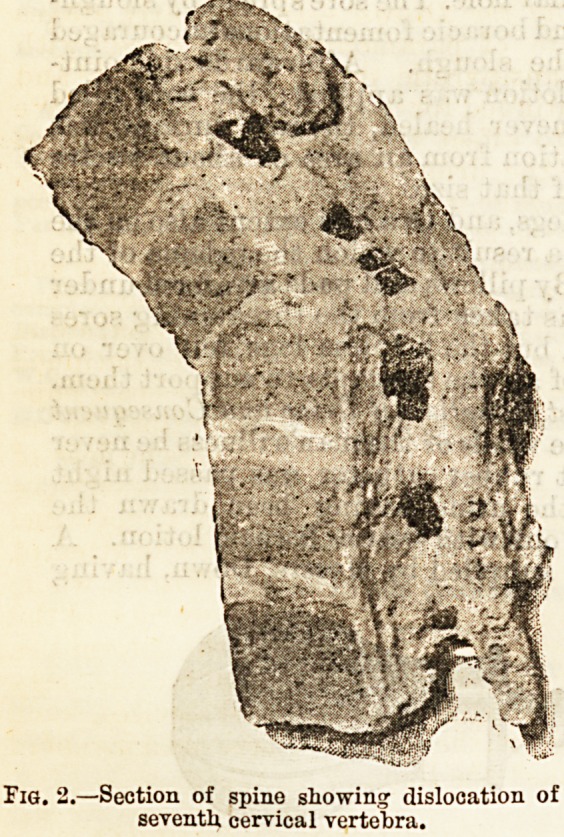


**Fig. 3. f3:**